# Extended-spectrum β-lactamase-producing *Escherichia coli* isolated from raw vegetables in South Korea

**DOI:** 10.1038/s41598-020-76890-w

**Published:** 2020-11-12

**Authors:** Jihyun Song, Sung-Suck Oh, Junghee Kim, Jinwook Shin

**Affiliations:** 1 Department of Microbiology, Inha University College of Medicine, 100 Inha-ro, Nam-gu, Incheon, 22212 South Korea; 2 Incheon Research Institute of Public Health and Environment, Incheon, 22320 South Korea

**Keywords:** Antimicrobial resistance, Food microbiology

## Abstract

The increasing prevalence of oxyimino-cephalosporin-resistant Enterobacteriaceae has become a global concern because of their clinical impact on both human and veterinary medicine. The present study determined the prevalence, antimicrobial susceptibility, and molecular genetic features of extended-spectrum β-lactamase (ESBL)-producing *Escherichia coli* (ESBL-EC) isolates from raw vegetables. A total of 1324 samples were collected from two agricultural wholesale markets in Incheon, South Korea in 2018. The ESBL-EC strains were isolated from 0.83% (11/1324) samples, and all of them were resistant to ampicillin, piperacillin, cefazoline, cefotaxime, and nalidixic acid and yielded CTX-M-type ESBL, including CTX-M-14, CTX-M-15, CTX-M-55, CTX-M-27, and CTX-M-65. The isolates belonged to phylogenetic subgroups D (n = 5), A (n = 4), and B1 (n = 2). Multilocus sequence typing revealed nine known *E. coli* sequence types (STs), including ST10, ST38, ST69, ST101, ST224, ST349, ST354, ST2509, ST2847, and two new STs. Notably, ST69, ST10, ST38, and ST354 belong to the major human-associated extraintestinal pathogenic *E. coli* lineages. Our results demonstrate that ESBL-producing multidrug-resistant pathogens may be transmitted to humans through the vegetable intake, highlighting the importance of resistance monitoring and intervention in the One Health perspective.

## Introduction

Gram-negative bacteria produce extended-spectrum β-lactamases (ESBLs) that are primarily responsible for resistance to oxyimino-cephalosporins such as cefotaxime. Among the ESBLs, the plasmid-mediated CTX-M-type enzymes have become predominant worldwide^1,2^. Based on the sequence identity of amino acids, five distinct groups of CTX-M-type enzymes (CTX-M-1, -2, -8, -9, and -25 groups) and more than 214 CTX-M-type enzymes have been reported (ftp://ftp.ncbi.nlm.nih.gov/pathogen/Antimicrobial_resistance; accessed January 24, 2020). In South Korea, many studies have been reported on ESBL-producing *Escherichia coli* strains (ESBL-ECs) in hospital and community settings since the first identification of CTX-M-14 in clinical isolates of *E. coli*, *Shigella sonnei*, and *Klebsiella pneumoniae* in 2001^3^. According to the national antimicrobial resistance surveillance system, the resistance rates for cefotaxime in clinical *E. coli* isolates increased gradually from 29% in 2013 to 35% in 2017^4,5^. Among the clinical isolates of ESBL-ECs, CTX-M-14 was the dominant type of ESBLs, and sequence type (ST) 131 accounted for more than 20% of them^6–11^. The prevalence of *E. coli* producing CTX-M-type ESBLs in healthy Korean adults was 8.3% in 2014^12^.

Besides humans, ESBL-ECs have been isolated from livestock, food, the environment, and other non-human sources^13–16^. It has been reported that animals can be a primary reservoir of ESBL-ECs^17^ and that foods may play a role in the dissemination of resistance to humans through the food chain^18–20^. For this reason, several developed countries have implemented antimicrobial resistance monitoring programs for food products, as well as in humans and animals based on the One Health approach^21^. A number of studies conducted in South Korea have also documented the prevalence of ESBL-ECs in multiple sectors, including livestock (chickens, 94.1%; pigs, 69.5%; cattle, 7.0%)^22^, imported meat (1.1%)^23^, companion animals (44.7%)^24^, ready-to-eat sprouts (3.3%)^25^, and river water (2.5%)^26^. Despite posing a high risk of transmission to humans through direct intake, few studies have been conducted on vegetable ESBL-ECs, so the available data is limited. Here, we investigated the prevalence, antimicrobial susceptibilities, responsible genes, and clonal lineages of ESBL-ECs isolated from raw vegetables in South Korea according to a nationwide surveillance program.

## Results

### Prevalence and antimicrobial susceptibility

**Figure 1:**
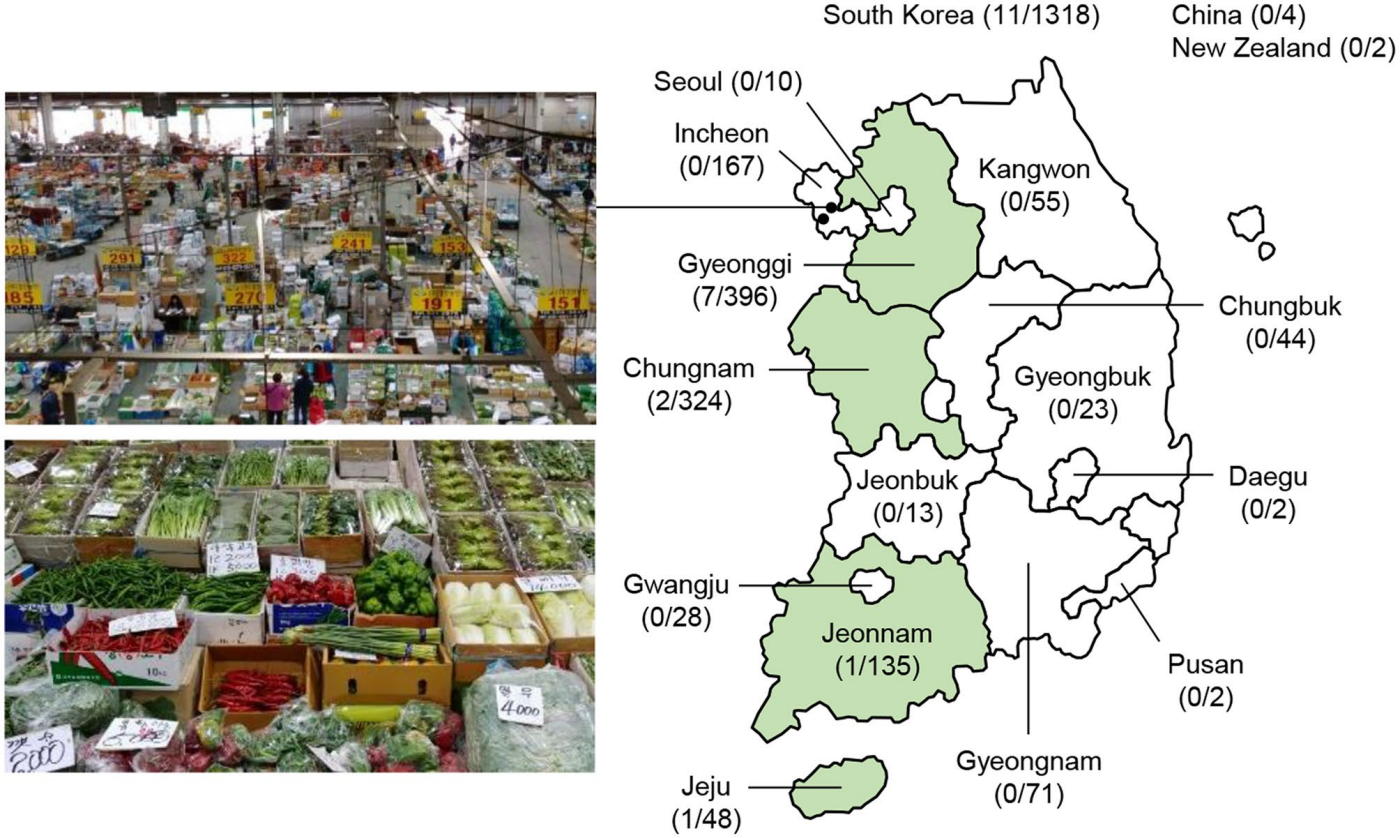
National wide distribution of cefotaxime-resistant *E. coli* isolates from vegetables. The black circle and green area represented the location of the wholesale market for agricultural products from which vegetables were collected and the production site of ​​them from which resistance was detected, respectively. The map was generated by using software program Microsoft PowerPoint 2016.

Among 1324 raw vegetable samples, a total of 11 non-duplicate cefotaxime-resistant ESBL-ECs were recovered from the stem (5/170, 2.94%) and leafy (6/879, 0.68%) vegetables (Fig. 1 and Table 1). No ESBL-ECs were isolated from the fruit and root types of vegetables. Antimicrobial susceptibility testing showed that all eleven isolates were resistant to ampicillin, piperacillin, cefazoline, cefotaxime, and nalidixic acid but susceptible to amikacin, ertapenem, imipenem, meropenem, tigecycline, and colistin (Table 1). The non-susceptibility rates for trimethoprim–sulfamethoxazole, aztreonam, ciprofloxacin, tetracycline, chloramphenicol, and gentamicin were 81.8%, 81.8%, 72.7%, 72.7%, 54.5%, and 45.5%, respectively. All of the isolates showed multidrug-resistant (MDR) phenotypes.

**Table 1:**
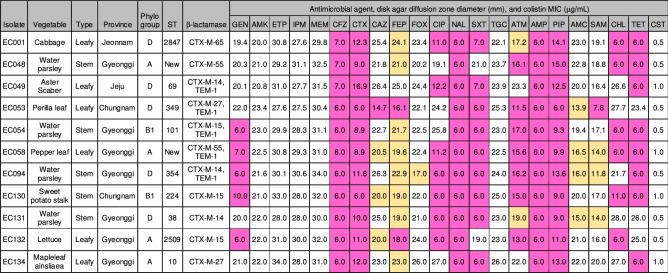
Characteristics of 11 cefotaxime-resistant *E. coli* isolates from vegetables.

The colors of pink and yellow indicated resistant and intermediate resistant to corresponding agents, respectively. The allele codes of the two new STs were *adk *(6), *fumC* (27), *gyrB* (4), *icd* (New), *mdh* (8), *purA* (8), and *recA* (6) for EC048 and *adk* (10), *fumC* (11), *gyrB* (5), *icd* (10), *mdh* (11), *purA* (8), and *recA* (6) for EC058. ST, sequence type; MIC, minimum inhibitory concentration; GEN, gentamicin; AMK, amikacin; ETP, ertapenem; IPM, imipenem; MEM, meropenem; CFZ, cefazolin; CTX, cefotaxime; CAZ, ceftazidime; FEP, cefepime; FOX, cefoxitin; CIP, ciprofloxacin; NAL, nalidixic acid; SXT, trimethoprim–sulfamethoxazole; TGC, tigecycline; ATM, aztreonam; AMP, ampicillin; PIP, piperacillin; AMC, amoxicillin–clavulanic acid; SAM, ampicillin–sulbactam; CHL, chloramphenicol; TET, tetracycline; CST, colistin.

### Characterization of β-lactamase genes

All of the isolates carried either *bla*_CTX-M_ group 1 (5/11, 45.5%) or *bla*_CTX-M_ group 9 (6/11, 54.5%) genes, including *bla*_CTX-M-15_ (n = 3), *bla*_CTX-M-14_ (n = 3), *bla*_CTX-M-55_ (n = 2), *bla*_CTX-M-27_ (n = 2), and *bla*_CTX-M-65_ (n = 1) (Table 1). The *bla*_CTX-M-27_ and *bla*_CTX-M-65_ were found only in the leafy vegetable isolates. Five isolates co-carried the non-ESBL gene *bla*_TEM-1_.

### Phylogenetic groups and MLST

**Figure 2:**
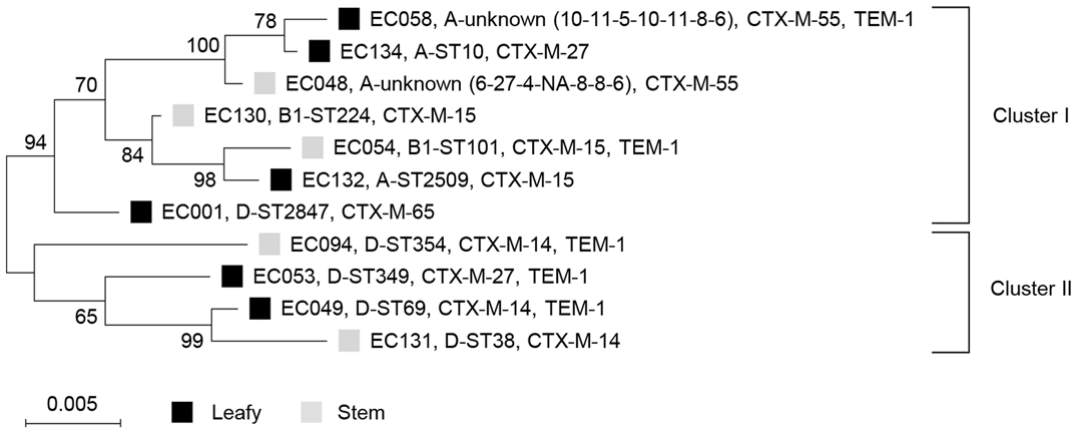
Phylogenetic tree of cefotaxime-resistant *E. coli* isolates from leafy and stem vegetables. Black and gray squares represented leafy and stem vegetables, respectively. Bootstrap support percentages were indicated in the different branches. Scale bar represented the genetic distance. Allele codes (*adk*-*fumC*-*gyrB*-*icd*-*mdh*-*purA*-*recA*) for new STs were indicated. NA, not assigned.

The phylogenetic analysis revealed that the ESBL-ECs belonged to subgroups D (5/11, 45.5%), A (4/11, 36.4%), and B1 (2/11, 18.2%) (Table 1). Two isolates belonging to subgroup B1 were detected only in stem vegetables. MLST analysis demonstrated that all of the isolates were assigned to different STs, including ST10, ST38, ST69, ST101, ST224, ST349, ST354, ST2509, ST2847, and two new STs. The allele codes for the new STs differed from ST6764 (allele code 10-11-5-10-12-8-6) by the *mdh* locus, ST1251 (6-27-4-10-8-8-6) and ST4967 (6-27-4-350-8-8-6) by the *icd* locus (Fig. 2). The MLST-based phylogenetic tree showed two major clusters, among which Cluster I comprised 85.7% commensal subgroups A and B1, and Cluster II was comprised only the virulent subgroup D (Fig. 2). There was no distributional difference between the clusters in leafy and stem vegetable isolates.

## Discussion

Fresh produce is usually consumed raw or not properly heated. Consequently, a considerable portion of recent foodborne outbreaks has been attributed to fresh produce contaminated by pathogens^27^. Likewise, antimicrobial resistance can readily spread to humans through the vegetables contaminated with resistant bacteria. ESBL-ECs have also been found in vegetables, principally ready-to-eat vegetables^19,25^ and raw vegetables^28–32^. In this study, we isolated the cefotaxime-resistant ESBL-ECs from eleven (0.83%) of 1324 raw vegetables, indicating a lower prevalence than that documented in a previous report on ready-to-eat sprouts (3/91, 3.3%) in South Korea^25^. The prevalence was about four times higher in the stem-type (2.94%) than the leafy-type samples (0.68%), but no isolates were detected in fruit and root types.

All 11 ESBL-ECs from vegetables in this study were MDR strains with resistance to ampicillin, piperacillin, cefazoline, cefotaxime, and nalidixic acid. Similarly, resistance to ampicillin, cefazoline, and cefotaxime were also reported on livestock and human ESBL-ECs in South Korea^8,22^. In addition, all of the vegetable isolates were susceptible to amikacin and tigecycline, which was consistent with previous reports in clinical isolates^33,34^.

Global epidemiology indicates that *bla*_CTX-M-15_ is the most prevalent ESBL gene worldwide^2^. Besides South Korea, however, *bla*_CTX-M_ group 9 (especially *bla*_CTX-M-14_) variants are dominant in China, South-East Asia, Japan, and Spain^2,7,8^. *bla*_CTX-M-27_ has been reported as the most common *bla*_CTX-M_ genotype in *E. coli* among patients in Vietnam^35^. All of the isolates from vegetables in this study harbored various *bla*_CTX-M_ genes, which include *bla*_CTX-M-14_, *bla*_CTX-M-15_, *bla*_CTX-M-55_, *bla*_CTX-M-65_, and *bla*_CTX-M-27_, representing the most common ESBL types worldwide. The presence of *bla*_CTX-M-55_, *bla*_CTX-M-65_, and *bla*_CTX-M-27_ has also been noted in *E. coli* isolated from humans, animals, and retail meat in South Korea^7,8,22,24,36–38^ as well as in neighboring countries, including China^39, 40^, Japan^41,42^, and Vietnam^43^. Despite the small number of *E. coli* isolates from different types of vegetables, the ESBL genotypes have been reported to be geographically distinct. The main types are *bla*_CTX-M-15_ in the Dominican Republic^30^, India^30^, and Ecuador^28^, *bla*_CTX-M-14_ in South Africa^31^, *bla*_CTX-M-55_ in Thailand^30^, and *bla*_CTX-M-65_ in Vietnam^30^. In South Korea, *bla*_CTX-M-55_ and *bla*_CTX-M-14_ were found in *E. coli* isolates from ready-to-eat sprouts between 2012 and 2013^25^, and *bla*_CTX-M-55_ was also detected in the colistin-resistant *E. coli* ST10 from lettuce in 2018^44^. In particular, the population of ESBL-ECs harboring *bla*_CTX-M-55_ has noticeably increased in both humans and animals in China^45,46^ and Vietnam^43^. In this study, we also identified *bla*_CTX-M-55_-harboring ESBL-ECs from 18.2% (2/11) of raw vegetables. Given that CTX-M-55 is a variant of CTX-M-15 with a single amino acid substitution of A77V^47^, our results and those of previous investigators suggest that CTX-M-55 may be replacing CTX-M-15, particularly in Asia.

*E. coli* phylogenetic groups A and B1 are typically commensal strains, whereas the B2 and D groups are mainly extraintestinal virulent strains^48^. In our study, the proportions were comparable between these commensal (6/11, 54.5%) and more virulent (5/11, 45.5%) lineages. MLST analysis revealed 11 different STs for the 11 ESBL-ECs in this study. Interestingly, four isolates (4/11, 36.4%) belonging to ST69, ST354, ST38, and ST10 were among the top 20 global extraintestinal pathogenic *E. coli* (ExPEC) lineages^49^, which are responsible for the majority of extraintestinal diseases, such as urinary tract infection, sepsis, and neonatal meningitis, and may have food animal sources^50^. These *E. coli* STs have been also observed in humans and livestock in South Korea^9,22,51^. The *bla*_CTX-M-14_-harboring *E. coli* ST38 and ST69 were reported in vegetables originating from the Dominican Republic^30^, India^30^, and China^52^. The *E. coli* ST10 strains producing CTX-M-type ESBL were often detected in vegetables^14,44,53^. To the best of our knowledge, this is the first report of ESBL-EC ST354 in vegetables. Furthermore, multiple ESBL-ExPEC clones, including ST131, ST405, ST410, and ST393, have been detected in raw vegetables^28,30^. These results suggest the potential roles of vegetables as the reservoir and propagator for ESBL-ExPEC lineages, which increases the threat of human health as a result of direct consumption.

It has been proposed that ESBL genes, plasmids, and strains may circulate among humans, animals, and the environment^13,54,55^. Notably, livestock functions as a primary reservoir of ESBL producers^17^, and the manure of animal feces contaminated with ESBL-ECs can be recycled as organic fertilizer during vegetable production^56^. In addition, ESBL-ECs have been found abundantly in waterbodies, such as surface water, irrigation water, and wastewater^57–59^, which can also be used in agricultural fields. Fecal bacteria can survive for a long time in soil, manure, and water^60^. Thus, fresh vegetables may have acquired resistance through direct or indirect contact with inoculum sources from the contaminated environment.

In conclusion, our findings showed the low prevalence rate of ESBL-ECs in vegetables but the presence of ESBL-ExPECs (ST10, ST38, ST69, and ST354) in stem and leafy, raw vegetables. In particular, the vegetables contaminated with human-associated MDR-ExPEC clones may give rise to further public health concerns and cause epidemics worldwide. Thus, our study emphasizes the importance of intensive monitoring and intervention for antimicrobial resistance from the perspective of the One Health approach, encompassing humans, animals, food produce, and the environment.

## Methods

### Study design

A total of 1324 raw vegetables, including 879 leafy, 236 fruit, 170 stem, 37 root, and two other types of vegetables, were collected from the Incheon’s two largest agricultural wholesale markets (Sam-San and Guwol markets) between February and October 2018. The countries of origin were South Korea (1318 samples), China (4 samples), and New Zealand (2 samples) (Fig. 1). The samples (30–60 g) were homogenized in a tenfold volume of *E. coli* (EC) broth (Difco Laboratories, Detroit, MI, USA) for 2 min and incubated at 37 °C overnight. Next, 1 ml of enriched media was added to 9 ml of Tryptone Soya Broth (Oxoid, Basingstoke, UK) supplemented with 0.4 g/ml vancomycin (Wako Pure Chemical Industries, Osaka, Japan), followed by incubation at 37 °C for 4 h. The enrichment was streaked on MacConkey agar containing 2 μg/ml cefotaxime. A non-duplicate colony of pink or reddish hue was picked and further grown on CHROMagar ESBL (CHROMagar, Paris, France) to obtain pure cultures. The *E. coli* species of isolates was determined using matrix-assisted laser desorption ionization-time of flight mass spectrometry (Bruker Daltonik GmbH, Bremen, Germany) with score values ≥ 2.0. To confirm ESBL production, the double-disk synergy test was performed using amoxicillin–clavulanic acid (20/10 μg), cefotaxime (30 μg), ceftazidime (30 μg), and cefepime (30 μg) disks.

### Antimicrobial susceptibility testing

Antimicrobial susceptibilities for 22 agents from 15 classes were analyzed by the disk diffusion method on Mueller–Hinton agar (Difco Laboratories) using 6-mm antibiotic disks (Oxoid). The following antimicrobial compounds (disk load) were assessed: gentamicin (GEN 10 μg), amikacin (AMK 30 μg), ertapenem (ETP 10 μg), imipenem (IPM 10 μg), meropenem (MEM 10 μg), cefazolin (CFZ 30 μg), cefotaxime (CTX 30 μg), ceftazidime (CAZ 30 μg), cefepime (FEP 30 μg), cefoxitin (FOX 30 μg), ciprofloxacin (CIP 5 μg), nalidixic acid (NAL 30 μg), trimethoprim–sulfamethoxazole (SXT 1.25/23.75 μg), tigecycline (TGC 15 μg), aztreonam (ATM 30 μg), ampicillin (AMP 10 μg), piperacillin (PIP 100 μg), amoxicillin–clavulanic acid (AMC 20/10 μg), ampicillin–sulbactam (SAM 10/10 μg), chloramphenicol (CHL 30 μg), and tetracycline (TET 30 μg). For the colistin (CST) susceptibility, the minimum inhibitory concentration was determined by the broth microdilution method using the Sensititre system (Thermo Fisher Scientific, Waltham, MA, USA). The phenotypes of resistance, intermediate resistance, and susceptibility were interpreted in accordance with the guidelines of the Clinical and Laboratory Standards Institute document M100-S27^61^, except for tigecycline breakpoints, which were interpreted based on the European Committee on Antimicrobial Susceptibility Testing breakpoint tables version 7.1^62^. Multidrug resistance (MDR) was defined as non-susceptibility to at least one antimicrobial agent of three or more classes^63^. *E. coli* ATCC 25922 was used as control strain.

### β-Lactamase genotyping

The pure DNAs of ESBL-ECs were extracted using the G-spin Total DNA Extraction Kit (iNtRON Biotechnology, Seoul, Korea) according to the manufacturer’s protocol. To identify β-lactamase (*bla*) genes belonging to *bla*_CTX-M-1_, *bla*_CTX-M-2_, *bla*_CTX-M-9_, *bla*_CTX-M-25_, *bla*_TEM_, and *bla*_SHV_, PCR was performed, as previously described^22^. After amplicon sequencing, the resultant sequences were compared with those of the β-lactamase genes in the GenBank database using the NCBI Basic Local Alignment Search Tool (BLAST) (https://blast.ncbi.nlm.nih.gov/Blast.cgi).

### Phylogenetic analyses

The isolates were assigned to phylogenetic groups A (*chuA*^−^, TspE4C2^−^), B1 (*chuA*^−^, *yjaA*^−^, TspE4.C2^+^), B2 (*chuA*^+^, *yjaA*^+^), and D (*chuA*^+^, *yjaA*^−^) by a PCR-based assay as previously reported^64^. To determine *E. coli* sequence types (STs), the sequences of seven conserved housekeeping genes (*adk*, *fumC*, *gyrB*, *icd*, *mdh*, *purA*, and *recA*) were analyzed by multilocus sequence typing (MLST) in accordance with the EnteroBase protocol and database (https://enterobase.warwick.ac.uk; https://enterobase.readthedocs.io/en/latest/)^65^. The maximum-likelihood phylogenetic tree was constructed based on the MLST of the seven housekeeping genes using MEGA X (https://www.megasoftware.net/) with a bootstrap analysis of 1000 replicates.

## Data Availability

All data analyzed during this study are included in this published article.
